# Temporal and spatial summation of laser heat stimuli in cultured nociceptive neurons of the rat

**DOI:** 10.1007/s00424-022-02728-1

**Published:** 2022-07-22

**Authors:** Elisabeth Jubileum, Uta Binzen, Rolf-Detlef Treede, Wolfgang Greffrath

**Affiliations:** 1 Department of Neurophysiology, Mannheim Center for Translational Neuroscience (MCTN), Medical Faculty Mannheim, Heidelberg University, Ludolf-Krehl-Str. 13-17, 68167 Mannheim, Germany; 2 Present Address: Department of Child and Adolescent Psychiatry, Psychotherapy and Psychosomatics, Rheinhessen Clinics, Hartmühlenweg 2-4, 55122 Mainz, Germany; 3 Department of Child and Adolescent Psychiatry and Psychotherapy, University Medical Center of the Johannes Gutenberg University, Langenbeckstraße 1, 55131 Mainz, Germany; 4 Department of Cardiovascular Physiology, European Center for Angioscience (ECAS), Medical Faculty Mannheim, Heidelberg University, Mannheim, Germany

**Keywords:** Heat pain, Nociception, Transduction, TRPV, Calcium imaging

## Abstract

We studied the efficacy of a near-infrared laser (1475 nm) to activate rat dorsal root ganglion (DRG) neurons with short punctate radiant heat pulses (55 µm diameter) and investigated temporal and spatial summation properties for the transduction process for noxious heat at a subcellular level. Strength-duration curves (10–80 ms range) indicated a minimum power of 30.2mW for the induction of laser-induced calcium transients and a chronaxia of 13.9 ms. However, threshold energy increased with increasing stimulus duration suggesting substantial radial cooling of the laser spot. Increasing stimulus duration demonstrated suprathreshold intensity coding of calcium transients with less than linear gains (Stevens exponents 0.29/35mW, 0.38/60mW, 0.46/70mW). The competitive TRPV1 antagonist capsazepine blocked responses to short near-threshold stimuli and significantly reduced responses to longer duration suprathreshold heat. Heating 1/3 of the soma of a neuron was sufficient to induce calcium transients significantly above baseline (*p* < 0.05), but maximum amplitude was only achieved by centering the laser over the entire neuron. Heat-induced calcium increase was highest in heated cell parts but rapidly reached unstimulated areas reminiscent of spreading depolarization and opening of voltage-gated calcium channels. Full intracellular equilibrium took about 3 s, consistent with a diffusion process. In summary, we investigated transduction mechanisms for noxious laser heat pulses in native sensory neurons at milliseconds temporal and subcellular spatial resolution and characterized strength duration properties, intensity coding, and spatial summation within single neurons. Thermal excitation of parts of a nociceptor spread via both membrane depolarization and intracellular calcium diffusion.

## Introduction

Noxious heat rapidly induces heat-evoked currents (*I*_heat_) and calcium transients in a subpopulation of small DRG neurons [5, 9–11, 18–20, 23, 30, 41]. Almost all of these studies used stimulation with heated extracellular solution, which is at least one order of magnitude slower than the transduction process itself. Early human laser-evoked potentials (LEP) and monkey electrophysiology studies had already suggested a rapid transduction mechanism for noxious heat based on rapid laser heating [39]. Therefore, in the last years, infrared lasers with a high rate of temperature change were also used to apply heat stimuli to dorsal root ganglion neurons or transfected human embryonic kidney cells (HEK293, [11, 16, 28, 44]). Greffrath and colleagues [11] induced heat-evoked currents (*I*_heat_) in dorsal root ganglion neurons by laser pulses of 980 nm wavelength. They assumed that *I*_heat_ was mediated by the capsaicin-receptor TRPV1 [4] due to the temperature threshold (42 °C) and the capsaicin sensitivity of the laser-sensitive neurons as well as tachyphylaxis of the laser-induced *I*_heat_. The reduction of *I*_heat_ induced with the same laser type by the competitive vanilloid receptor antagonist capsazepine (CPZ) further confirmed this assumption [16]. Due to the low absorption in water of this 980 nm laser, the power required in both studies was rather high (11 W [11]; 0.3 to 4.5 W [16]).

By using a longer wavelength near a water absorption peak (1460 nm) Yao and colleagues [45] were able to produce temperature jumps within milliseconds (23 to 53 °C within 0.75 ms). They analyzed the activation and deactivation kinetics of TRPV1 channels, which were heterologously expressed in HEK293 cells [45] and confirmed an activation threshold of TRPV1 near 40 °C. The activation rate and the steady state-currents increased strongly with steeply rising temperatures. A very fast activation was shown with a time constant of 6 ms (in comparison, the time constant of activation of the same channels by capsaicin was 110 ms).

All these laser systems used a glass fiber close to the cell under study to transmit stimulus energy to the cell. For wavelengths with low absorption in water, positioning is uncritical, but very high power is needed to deposit enough energy on the cell. For wavelengths with high absorption in water, efficient cell stimulation is possible, but the positioning of the glass fiber becomes critical. In this study, a setup to apply near-infrared laser pulses (1475 nm) for live-cell imaging applications without interference by glass fibers or extracellular solution was developed to stimulate native rat dorsal root ganglion neurons (DRG) with ultrashort punctate laser heat pulses and subcellular spatial accuracy. Cells were stimulated directly through a glass plate without passing the extracellular solution. Stimulus–response curves for three different laser powers as well as the minimal stimulus durations and threshold energies needed to induce calcium transients were determined. For the first time, only parts of a single neuron could be heated with this setup and thus, the sizes of calcium transients were identified as a function of the size of the cell area heated. Evidence for spatial summation within the branching region of distal terminals of nociceptive primary afferents had previously been found in monkeys [37].

We now used punctate laser heat pulses to investigate the properties of laser heat transduction in native sensory neurons at milliseconds temporal and subcellular spatial resolution. The following specific aims were addressed: (i) characterization of temporal and spatial summation processes within the somata of nociceptive DRG neurons, (ii) assessment if calcium transients can spread to unstimulated parts of the soma, and if so, if this spread is via diffusion or some other mechanism.

## Materials and methods

### Preparation of acutely dissociated cells

The neurons were prepared and dissociated as described in previous studies [9]. Adult Sprague–Dawley rats (160 ± 60 g) of either sex (15 female, 32 male) were deeply anesthetized with diethyl ether and rapidly decapitated. This method was in accordance with German national law and the principles of animal welfare and was approved by the local representative for animal care and use of the University. All efforts were made to minimize the suffering of animals as well as the number of rats used for the experiments. The spine was removed, chilled at 4 °C in F12-Dulbecco’s modified Eagle`s medium (Sigma-Aldrich, Darmstadt, Germany; adjusted to pH 7.4 by NaOH) containing 26 mM NaHCO_3_ (Merck, Darmstadt, Germany) and 100U/ml penicillin and 100 µg/ml streptomycin (Sigma-Aldrich). The F12 medium was equilibrated with 95% O_2_ / 5% CO_2_ throughout the whole preparation and dissociation procedure. Neurons were enzymatically dissociated at 37 °C with collagenase CLSII (5 mg/ml, 45–60 min; Biochrom AG, Berlin, Germany) dissolved in the F12 medium on a shaker. After rinsing the tissue three to four times in F12 medium, neurons were mechanically triturated using fire-polished Pasteur pipettes. Cell suspension was centrifuged (800 g for 5 min) through Neurobasal-A Medium (Invitrogen, Karlsruhe, Germany) containing 15% bovine serum albumin (BSA; Roth, Karlsruhe, Germany) to purify the neurons. Centrifugation was repeated at 400 g for 5 min in Neurobasal-A Medium to pellet the purified neurons. Neurobasal-A Medium was carefully removed and 1 ml of fresh Neurobasal-A Medium was added to dispense the pellet of cells. Neurons were plated on microscope cover glasses transparent to UV and NIR light (diameter: 22 mm; Roth). To improve adhesion, cover glasses were coated with Poly-L-Lysin (10 µg/ml; Sigma-Aldrich). Neurons were stored for 1 to 2 h at 37 °C in a humidified 5% CO_2_ atmosphere to allow proper adherence to the cover glasses. Neurobasal-A Medium with 2% heat-inactivated horse serum, 2 mM L-glutamine (PAA Laboratories GmbH, Cölbe, Germany), penicillin and streptomycin, B-27 supplement (1:50; Invitrogen), and nerve growth factor (50 ng/ml; NGF murine, Invitrogen) were added to the neurons, which were stored overnight.

### Intracellular calcium measurements

After a minimum resting period of 1 to 2 h, neurons were transferred into an extracellular solution containing (in mM) 137.6 NaCl, 5.4 KCl, 0.5 MgCl_2_, 1.8 CaCl_2_, 5 glucose, and 10 HEPES (pH 7.34; all salts purchased from Merck or Roth). Neurons were loaded for 1 to 1.5 h with the cell-permeable acetoxymethyl ester form of the fluorescent Ca^2+^-indicator FURA-2 (FURA-2-AM, 3 µM; Calbiochem, Merck) at room temperature in the dark. FURA-2-AM was prepared as 1 mM stock solution in dimethyl sulfoxide (Merck), stored at − 25 °C and diluted to 3 µM in extracellular solution. After loading, neurons were washed several times with extracellular solution to remove extracellular dye.

**Fig. 1:**
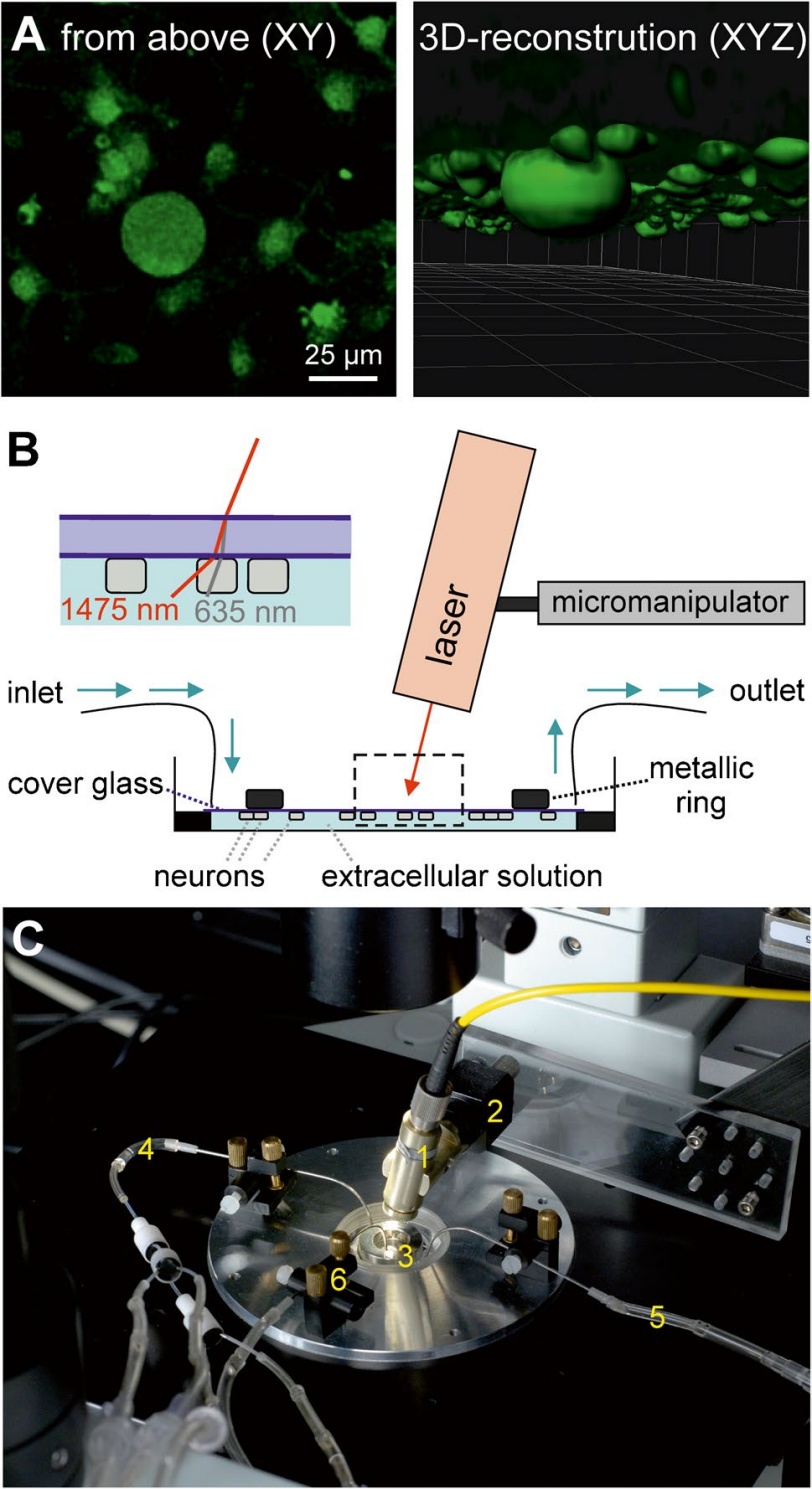
Experimental setup for investigation of diode laser-induced calcium transients in cultured nociceptive neurons of rats. Laser pulses were applied from above through the cover glasses directly to the neurons that adhere to the back of the cover glasses. Extracellular solution was perfused below the neurons through the small space between the cover glass and the bottom of the culture dish. **A** FURA-2 loaded cells as visualized in the usual XY-plane normally used in calcium imaging experiments (left) as well as after three-dimensional XYZ-reconstruction illustrating the cells hanging upside down from the cover glass (right). Note one mid-size DRG neuron surrounded by several smaller satellite cells. **B** Sketch of the experimental setup; dotted lines indicate magnified inset demonstrating the laser stimulation of hanging cells from above; red line indicates weaker refraction of the invisible NIR laser as compared to the visible pilot laser (grey line). **C** Photography of the experimental set-up. 1 collimator with glass fiber, 2 micromanipulator, 3 culture dish with cover glass, 4 inlet, 5 outlet, 6 extraction system to keep the surface of the cover glass dry

Cover glasses with adherent neurons were transferred upside down into a culture dish maintained at room temperature in an inverted microscope (IX 70, Olympus, Hamburg, Germany) that was equipped with a monochromator (Polychrome IV, TILL Photonics GmbH, Graelfing, Germany). For three-dimensional reconstruction of the hanging neurons, a multiphoton microscope with a super-Z galvo stage (TCS SP5 Mid System (UV–VIS-IR), Leica Microsystems, Mannheim, Germany) was used at appropriate two-photon excitation of FURA-2 loaded DRG neurons and Imaris 9 software (Bitplane AG, Zurich, Switzerland; Fig. 1). These measurements were essential to confirm that a neuron being investigated while hanging upside-down in fact displays similar diameters as compared to routine cultures of native cells (e.g., for identifying small-diameter nociceptive neurons, correctly) and to demonstrate that the major volume of the cell in this configuration is indeed located near to the surface where the laser is mainly absorbed—and not, e.g., a tubular-like hanging configuration.

Laser-heat pulses were applied from above through the cover glass directly to the neurons without passing the extracellular solution that was perfused below the neurons through the small space between the cover glass and the bottom of the culture dish (Fig. 1).

For fluorescence measurements of intracellular calcium, all images were taken with a CCD camera (Imago, TILL Photonics). The fluorescence dye in single neurons was sequentially excited at 340 and 380 nm wavelength and the emission light signals at 510 nm were digitized (1–25 frames s^−1^) and stored on a personal computer running TILLvisION 4.2. software (TILL Photonics). The ratio 340/380 nm of these fluorescence signals provides a relative measure of intracellular calcium concentration [13]. In the present study, only neurons with a maximum diameter of 32.5 µm were included.

In control experiments, fluorescent measurements were done in an extracellular or intracellular solution containing 30–50 µM FURA-2 to mimic the intracellular situation where calcium is low and FURA-2 is trapped and markedly enriched by intact cells by an order of magnitude or even more [26]. Changes in FURA-2-ratio 340/380 nm in response to laser stimulation were recorded within a round area with a diameter of 25 µm.

### Diode laser stimulation

A near-infrared (NIR; wavelength 1475 nm) diode laser stimulator (Laserdioden-Strahlquelle SK9-2001, Schaefter & Kirchhoff, Hamburg, Germany) was used for laser-heat application. The NIR radiation was coupled with a visible laser (635 nm) via a single-mode optic glass fiber. The fiber collimator that focused the two laser radiations was moved by a computer-controlled motorized micromanipulator (Sutter MP-285, Sutter Instruments, Novato, CA, USA) in 40-nm steps. Both laser radiations were collinear, but due to the different wavelengths their focal spots had different distances from the collimator; the distance between these two laser foci (930 µm) was used to adjust and focus the NIR radiation to a single neuron. The beam diameter to induce calcium transients was adjusted to 55 µm. With separated potentiometers, output power was manually regulated for both wavelengths. The maximum output power of the 1475 nm radiation was 100 mW and 1 mW of the 635 nm laser. The pilot laser power was kept constant at a maximum of 10 %. Stimulus duration was controlled with a personal computer. Absolute stimulus temperatures induced by the laser pulses were not systematically determined in this study; please refer to [28] for measurements of stimulus temperatures induced by those laser pulses of different intensities and durations.

### Threshold determination and stimulus–response functions

Thresholds for different laser powers were determined by varying stimulus duration in steps of a factor of two (1, 2, 4, …, 2048 ms). Interstimulus intervals (ISIs) were 30 s. The first stimulus duration that led to a specific cellular response was taken as the threshold value, provided the next longer duration (or a repetitive stimulus) also induced a specific response. This way, we also obtained at least two different responses of each neuron to analyze its suprathreshold intensity encoding properties. The 50%-threshold was defined by the stimulus duration and energy inducing a calcium transient in half of the neurons tested.

### Spatial mapping of cellular responsiveness

One advantage of laser heat stimulation is the possibility to apply localized, focused heat stimuli. We therefore tested which fraction of the surface of the neuron has to be heated to elicit laser-induced calcium transients. The laser stimulus (spot diameter = 55 µm, 70mW for 16 ms, 30 s ISI) was applied every 10 µm along a mapping axis (distance 140 µm; Fig. 7). The overlap between the laser spot and the neuron was calculated. In these experiments, maximum calcium transients were detected at a relative position 20 µm lateral to the pilot laser spot. This indicated a shift of the pilot laser spot versus the infrared laser spot of about 20 µm, probably due to the different refraction of the two wavelengths in the coverslip. In all experiments of the present study, the focusing of the infrared laser spot to the center of the neuron was performed considering this lateral shift (Fig. 1 and Fig. 7).

### Drug application

A solution exchanger which combined four solution reservoirs to one single outlet offered a continuous flow with the extracellular solution during the whole experiment. Thus, the application of two different drugs and their respective vehicles was possible within a single experiment. The flow of the solution was driven by gravity. Capsaicin (Sigma-Aldrich) was prepared as a concentrated stock solution in ethanol (Roth) and diluted to 10 µM in extracellular solution and applied for 30 − 75 s. CPZ (10 µM; dissolved in dimethyl sulfoxide, DMSO) and DMSO-vehicle-solution with a final concentration of 0.1% in extracellular solution were applied for 6 min (5 min before and 1 min after laser stimulation). This relatively high antagonist concentration—as compared to blocking chemical activation of the TRP-channel—was necessary because the weaker physico-chemical binding of the antagonist reduces its efficacy; the chosen concentration had been shown before to effectively reduce heat-responses in DRG neurons and TRPV1-expressing cells (see, e.g., [4, 36, 18, 9]). ISI was 10 minutes.

### Data analysis

Evaluation and statistical analysis were done using TILLvisION 4.2 Software (TILL Photonics), Axon pCLAMP 9.2/Clampfit (Molecular Devices, San Jose, California, USA), and EXCEL 2003 and 2013 (Microsoft). Time constant τ of the temperature rise and the temperature decay (mean ± sd) induced by near-infrared laser stimuli were determined indirectly using temperature-dependence of FURA-2-fluorescence ratio 340/380 nm in cell-free medium to estimate the temporal characteristics of the temperature changes induced by our laser pulses. Time constants were determined in control experiments with a high temporal resolution (25 Hz).

To characterize calcium transient of the neurons, the following parameters were used: Peak amplitude was quantified relatively to the average FURA-2-fluorescence ratio 340/380 nm over 10 s before any laser stimulation (“initial baseline”). Peak amplitude was presented as original traces and as mean ± SEM. An increase of FURA-2-fluorescence ratio 340/380 nm beyond 123% was defined as a true laser-induced calcium transient in neurons [9]. Prestimulus baseline (FURA-2-fluorescence ratio 340/380 nm over 5 s before current laser stimulation) was also quantified relatively to the initial baseline in some experiments, when rapid stimulus series lead to incomplete recovery and intracellular calcium accumulation (indicated as baseline). The change in ratio was calculated as a relative increase in ratio as compared to the initial baseline. The decay time of calcium transients was measured from 80% of the peak amplitude to recovery to 20% of the peak amplitude and was presented as median (minimum–maximum). Effects were statistically analyzed using Student’s t-test for paired and unpaired parametric data and Mann–Whitney U-test for unpaired nonparametric data. Due to improvement of the skewness of distribution all data were transferred to and analyzed in logarithmic space. Retransformed linear values were shown in the figures and text to improve readability. Distributions were tested by the Kolmogorov–Smirnov test. One-way ANOVA with LSD post hoc test was used to compare the effect of the different overlapping positions between neuron and laser-spot on the difference between peak amplitude or the prestimulus baseline. In all tests, *p* < 0.05 was considered significant.

## Results

### Specific laser heat responses in native nociceptive neurons

As confirmed by laser-scanning microscopy followed by 3-dimensional reconstruction, neurons investigated upside-down displayed typical roundish shapes as in usual culture (see Fig. 1A for a representative example) which allows to measure the diameter as usual. The upside-down arrangement was necessary to allow for near-infrared laser stimulation of the cell without passing through extracellular solution first which would have absorbed much of the laser energy.

**Fig. 2:**
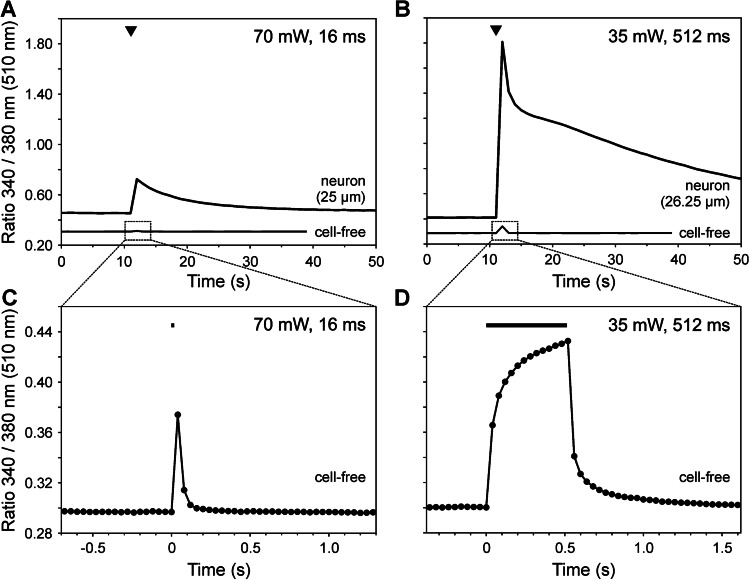
Laser heat stimulation rapidly induces short-lasting temperature changes and triggers longer-lasting calcium transients in rat DRG neurons**.** Representative examples of calcium transients (upper trace, “neuron”) and temperature waveforms (lower trace, “cell-free”) induced by short (**A** 70mW, 16 ms) and long laser pulses (**B** 35mW, 512 ms, indicated as arrowheads) recorded with identical specifications for comparison (1 Hz sampling rate). Calcium transients outlasted heat pulses by 10 s to 10 min. The temperature waveforms in (**C**) and (**D**) display spatially and temporally amplified temperature-dependent fluorescence changes in cell- and calcium-free extracellular solution at a sampling rate of 25 Hz (as indicated by the filled circles). **C** A fast rise and decline of the temperature were induced by short and high-intensity laser stimulation (70mW, 16 ms). The sampling rate used in cell calcium imaging experiments (**A**, **B**) was not high enough to detect these rapid temperature changes consistently. **D **When stimuli were applied at a lower intensity with a longer duration (35mW, 512 ms) temperature rose linearly within the first 80 ms of the laser pulse, then displayed an asymptotic slower increase and declined immediately by passive cooling when the laser was turned off

To illustrate properties of laser-induced calcium transients in small-diameter neurons hanging upside-down, we used two different laser pulses (Fig. 2):

1. Short near-threshold laser pulse (70mW, 16 ms; 1.1 mJ, *n* = 7)
2. Long suprathreshold laser pulse (35mW, 512 ms; 18 mJ, *n* = 8).

**Fig. 3:**
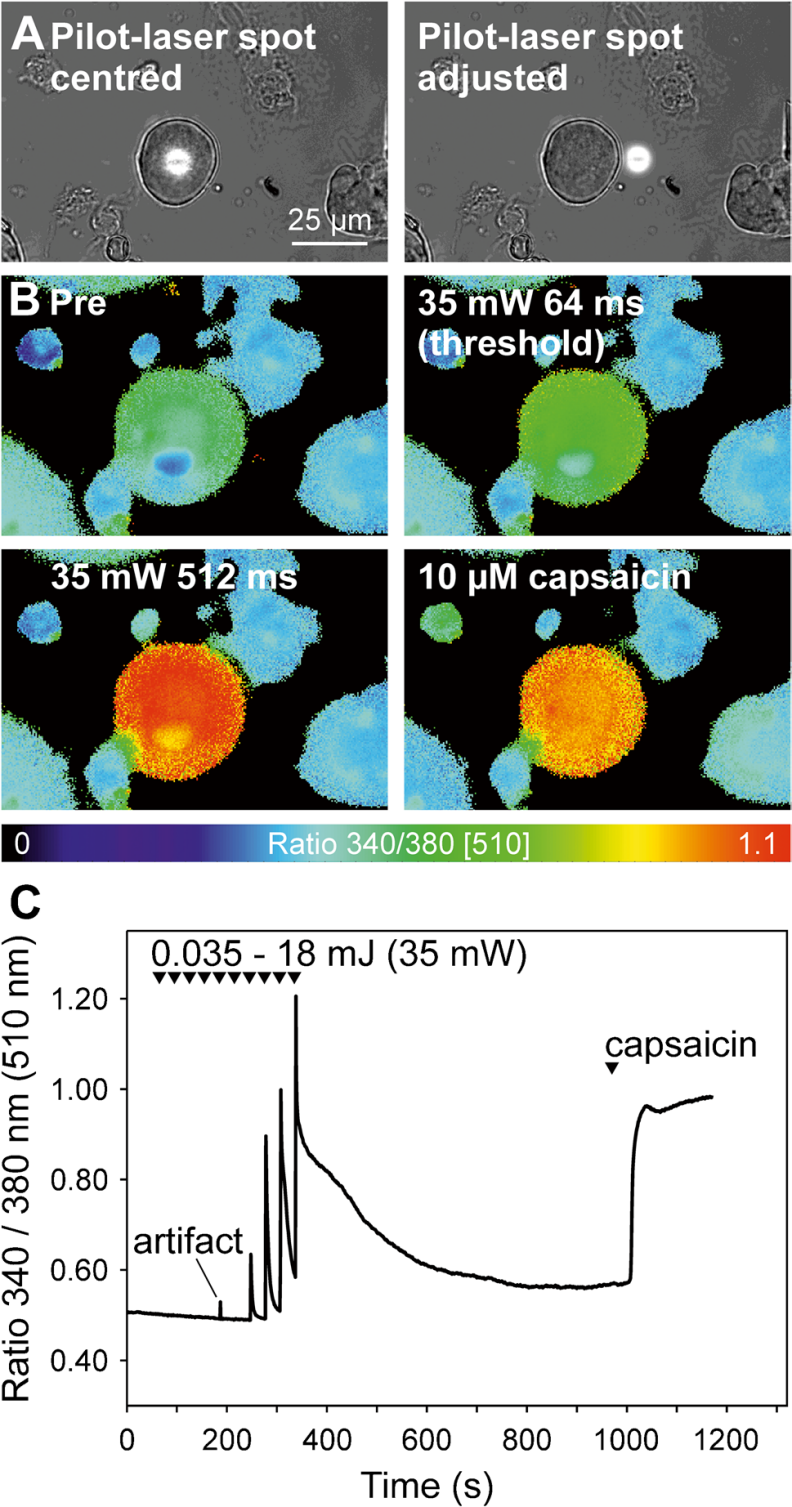
Laser-evoked calcium responses in nociceptive rat DRG neurons. **A** Bright-field image of a representative DRG neuron with the pilot laser centred to the middle of the neuron (left) and after adjusting by 20 µm lateral to the right to compensate for differing refraction of the two laser radiations used (compare Figs. 1 and 7). **B** False colour-coded ratiometric calcium imaging pictures exhibited the change in intracellular calcium in response to laser and chemical stimulation. When stimulated with 35mW, the capsaicin-sensitive neuron displayed a first significant calcium transient at a stimulus duration of 64 ms (2.2 mJ) and a stronger response to 512 ms (18 mJ). **C** Calcium changes over time from the neuron shown above (note the small stimulus “artifact” with a duration ≤ 2 s due to the temperature dependence of FURA-2 at 16 ms [0.56 mJ]). The responses rise regarding amplitude and duration at increasing stimulus durations and thus this neuron encoded stimulus intensity up to the maximum stimulus duration applied (512 ms = 18 mJ). Triangles mark laser pulses with a stimulus duration from 1 to 512 ms and one application of 10 µM capsaicin at the end of the experiment

Figure 2 (upper row) shows representative examples of cellular calcium transients induced by laser stimuli of 1.1 mJ (70mW for 16ms; Fig. 2A) and 18 mJ (35mW for 512 ms, Fig. 2B) that were recorded at an acquisition rate of 1 Hz. The laser-induced calcium transients met the previously published criterion for a specific cellular response (123% of baseline, [9]), which was based on mean plus 3 standard deviations of temperature artifacts of FURA-2 in cell- and calcium-free solution to rapid solution exchanges. When these control experiments were repeated with the much faster laser, heat-induced temperature artifacts at the normal sampling rate of 1 Hz were usually not seen at all—please refer to the traces “cell free” in Fig. 2A and Fig. 2 B as compared to the much larger responses seen in response to the same stimuli in “neurons”. Only occasionally, one single data point was above baseline that fully returned after 2 s at an acquisition rate of 1 Hz (for example see “artifact” in Fig. 3, bottom row).

These temperature waveforms of laser heat stimuli were determined more precisely at much higher acquisition rates—according to the Nyquist-Shannon-theorem at least 125 Hz and 4 Hz for 16 ms and 512 ms. Thereby, the temperature dependence of fluorescence could be used to estimate temporal heat stimulus characteristics. Increasing to an available sampling rate of 25 Hz (Fig. 2C and D) revealed that during the longer pulse, the temperature rose linearly only during the initial 80 ms and then leveled off towards a plateau (time constant τ of rise = 75 ± 10 ms), probably due to lateral temperature diffusion (Fig. 2D). The time constant τ of decay by passive cooling of the small heated volume was even significantly shorter (47 ± 14 ms, *p* < 0.01 versus rise, *n* = 7). Due to the limited sampling rate, the steep increase of the short laser pulses could not be determined exactly, but the slower recovery could (Fig. 2C).

The longest decay time of such temperature artifacts was 1.2 s (for 2048 ms laser stimulus duration, at a sampling rate of 1 Hz); hence, we accepted only responses with at least 2-s decay time as specific cellular responses. For 2048-ms stimulus duration (adequately recorded with a sampling rate of 1 Hz), the mean amplitudes of temperature artifacts at both 35 and 70mW were similar to the published data (103 and 108% of baseline), so we maintained the amplitude criterion (123% of baseline) and added decay time of at least 2 s as a second criterion for specific cellular responses [9].

In neurons, calcium rapidly rose during the laser pulse and started to decrease slowly immediately afterwards. Amplitudes were 158% (2.198 ± 0.024) at 1.1 mJ and 416%, and thus significantly larger, at 18 mJ (2.620 ± 0.037; *p* < 0.001, unpaired t-test). Nominal peak times of all calcium transients in response to laser stimuli were 2 s. Decay times of those induced by short laser pulses were at least 10 s (up to > 600 s; *n* = 7); in contrast, decay times of calcium transients induced by long laser pulses were at least 26 s (up to > 600; *n* = 8). Thus, calcium transients outlasted the duration of heat stimulation by 10 s to 10 min (compare Fig. 2). At long pulses, a biphasic time course was observed in half of the neurons, whereas the remaining as well as those induced by 1.1 mJ declined with a monophasic time course. Due to that large variation, however, differences in decay times of calcium transients failed to reach significance (*p* = 0.093, Mann–Whitney U-test).

### Sensitivity of DRG neurons to heating by infrared laser

Calcium transients were measured in 160 DRG neurons (diameter 25.8 ± 1.4, see Table 1); 84 of them were tested with laser stimuli of increasing energy by increasing stimulus duration to determine the threshold. All of these 84 neurons displayed calcium transients beyond the temperature dependence of the fluorescence dye FURA-2. Using the visible pilot laser, we aimed the laser beam precisely at one single DRG neuron (Fig. 3A, left) and corrected by 20 µm to the right to account for the different refraction of the laser beams (Fig. 3A, right). The neuron shown responded with heat-induced calcium transients to 35mW pulses of 64–512-ms duration and it encoded laser pulse intensity in this range of energies (2.2–18 mJ). At the end of the experiment, its response to 10 µM capsaicin revealed TRPV1 expression (Fig. 3B, C).

**Table 1:** Properties of the investigated neurons

	Numbers		Diameter		Baseline ratio	
	Heat ^+^	Heat ^−^	Heat ^+^	Heat ^−^	Heat ^+^	Heat ^−^
Threshold determination:						
35 mW	25	1	25.2 ± 1.3	25.0	0.48 ± 0.01	0.44
60 mW	19	3	25.2 ± 1.4	25.8 ± 1.4	0.45 ± 0.02	0.46 ± 0.1
70 mW	31	5	25.5 ± 1.1	27.1 ± 1.9	0.48 ± 0.01	0.46 ± 0.03
TRPV1 blockade:						
70mW, 16 ms	20		25.6 ± 1.0		0.44 ± 0.01	
35mw, 512 ms	20		26.1 ± 1.1		0.43 ± 0.01	
Spatial mapping:						
70mW, 16 ms (1 Hz)	16		26.9 ± 1.5		0.40 ± 0.01	
70mW, 16 ms (≥ 5 Hz)	15		25.8 ± 1.6		0.43 ± 0.01	
35mW, 512 ms (≥ 5 Hz)	5		27.0 ± 1.6		0.50 ± 0.03	

Properties of all neurons investigated with calcium microfluorimetry at a standard acquisition rate of 1 Hz (diameter: mean ± sd; baseline ratio: mean ± sem). Note that a classification into laser-sensitive (heat^+^) and laser-insensitive (heat^−^) neurons was only possible for the experiments that were done for threshold determination. The experimental series “spatial mapping (1 Hz)” and “TRPV1 blockade” only included laser-heat-sensitive neurons

Nine of the 84 neurons exhibited a different response pattern: they needed a relatively high laser energy to induce increases in intracellular calcium (4.60 ± 5.35 mJ, mean ± sd) and these increases were irreversible. Therefore, these nine neurons were considered laser-insensitive but probably nonspecifically affected or even damaged by laser-heat stimulation. Thus, the percentage of specifically laser-sensitive neurons was 89% (75/84) of the small neurons under investigation (diameter ≤ 32.5 µm).

### Activation of TRPV1 contributes to laser-evoked calcium transients

**Fig. 4:**
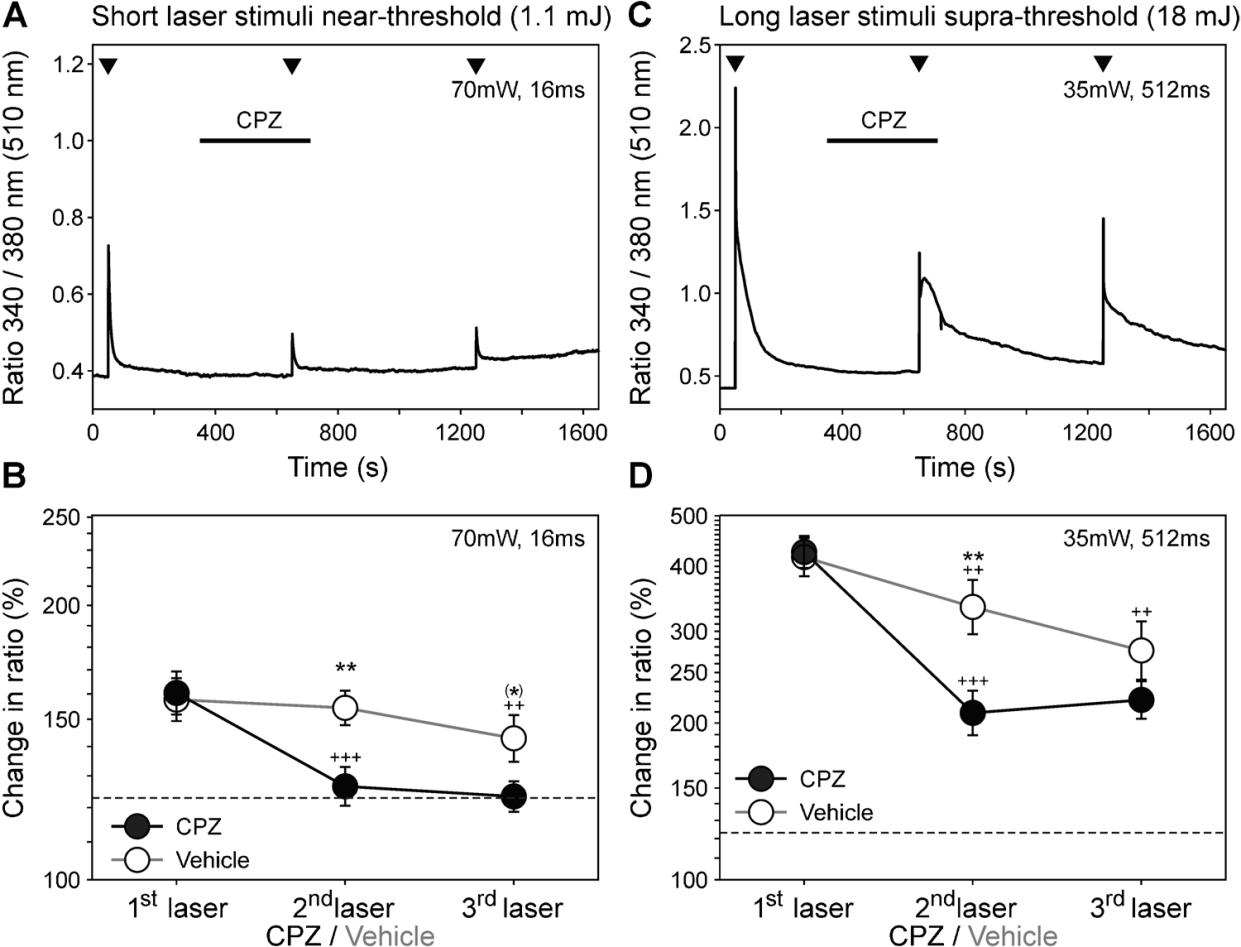
Laser-induced calcium transients are blocked by the competitive TRPV1 antagonist capsazepine (CPZ). **A** and **B** display short, near-threshold stimuli (70mW, 16 ms), **C** and **D** long, suprathreshold stimuli (35mW, 512 ms); ISI = 10 min. A significant, partially reversible reduction of calcium transients by CPZ (10 µM) was shown and, thus, an activation of TRPV1 by laser was proved. **A** and **C** show representative examples of neurons tested repeatedly with short and long laser pulses (arrowheads). **B** and **D** show statistics of all single neurons tested with CPZ (filled) or vehicle (open symbols). Dashed lines indicate the threshold for significant laser responses (123%). All data are given as mean ± sem, *n* = 13 and 7 for short stimuli with/without CPZ, and *n* = 12 and 8 for long stimuli. ^(^*^)^*p* = 0.06, **p < 0.01, Students unpaired t-test, CPZ versus vehicle; ^++^ *p* < 0.01 for long and short stimuli, ^+++^ *p* < 0.001 Students paired t-test, versus first stimulus

All four neurons sensitive to laser-heat stimulation were also sensitive to capsaicin, indicating expression of the vanilloid receptor TRPV1 (for example see Fig. 3). To assess whether these laser-induced calcium transients were mediated by the heat-sensitive TRPV1, we tested whether they were reduced by the competitive TRPV1 antagonist capsazepine (CPZ, 10 µM, Fig. 4).

When stimulated repetitively with short stimuli at an interstimulus interval of 10 min, neurons reliably responded to each pulse without any tachyphylaxis (first response increased to 158% [log_10_ 2.20 ± 0.02], second response to 154% [2.19 ± 0.02]; *p* = 0.617, Student’s paired t-test). Preincubating CPZ for 5 min reduced those specific laser heat responses from 160 to 127% (log_10_ 2.21 ± 0.02 to 2.10 ± 0.02; Student’s paired t-test *p* < 0.001 versus first; Fig. 4A, B), eight of 13 were eliminated completely, i.e., below an increase of 123%. In contrast, upon repeated laser stimulation of long duration, neurons displayed tachyphylaxis. In the vehicle group (*n* = 8), the first response was an increase to 416% (2.62 ± 0.04) whereas the second response was to 334% (2.52 ± 0.05; Student’s paired t-test *p* < 0.01). CPZ reduced those from an initial increase to 426% to 209% (2.63 ± 0.03 and 2.32 ± 0.04; Student’s paired t-test *p* < 0.001 versus first; Fig. 4C, D), whereupon only one of 12 was eliminated completely (i.e., < 123% [2.09]).

In summary, CPZ fully blocked heat responses near threshold—suggesting mediation by TRPV1—whereas those being suprathreshold were reduced by about 60% but not fully blocked which may indicate additional heat transduction mechanisms (cf. [28]).

The 15 neurons of the vehicle groups were the same neurons used for determining response criteria (see the “Specific laser heat responses in native nociceptive neurons” section). For each neuron, only the first specific laser-induced calcium response was analyzed to avoid effects of fatigue upon repetitive testing.

### Threshold energy as a complex function of stimulus duration

**Fig. 5:**
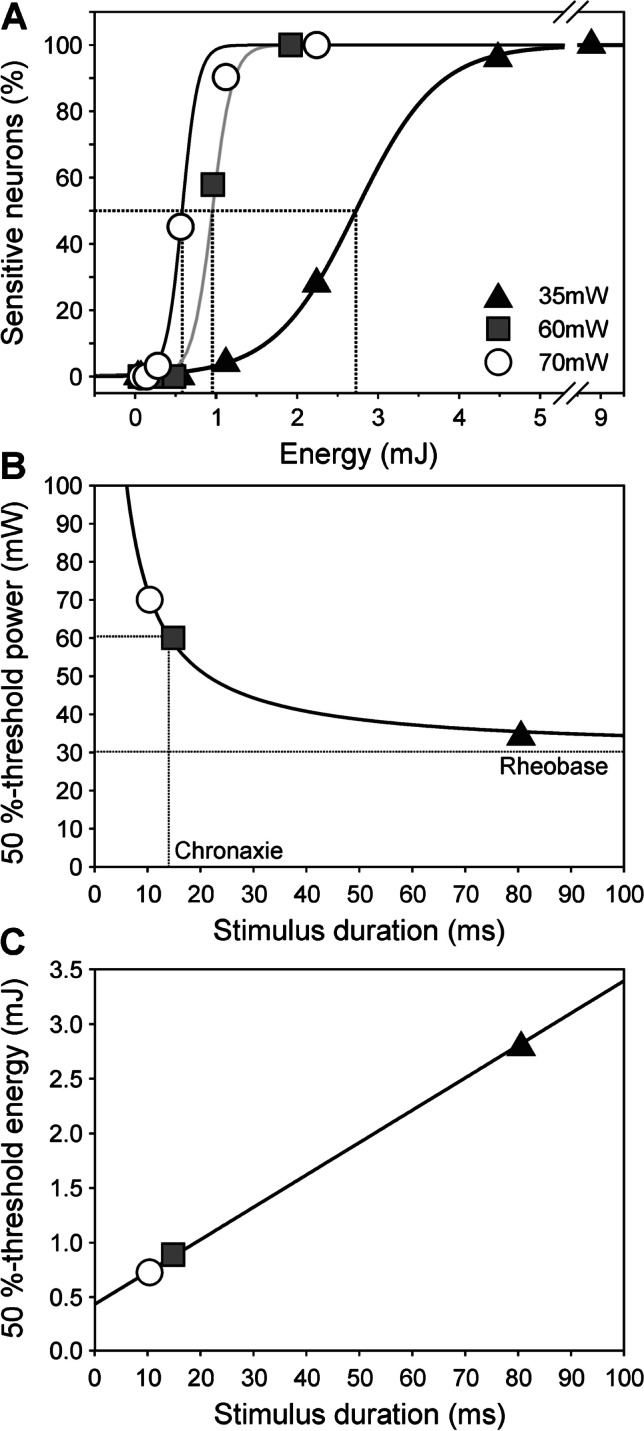
Laser-heat threshold is a non-linear function of laser energy. **A** 50%-threshold for induction of specific laser responses was defined as energy needed to induce significant calcium transients in 50% of the neurons and is indicated by dotted lines. Sigmoidal stimulus–response functions rose steeper at higher laser power (35mW: *n* = 25; 60mW: *n* = 19; 70mW: *n* = 31). **B** The threshold power declined with increasing stimulus duration as a hyperbolic function suggesting that energy (= power × duration) may be constant. Rheobase was extrapolated as 30.2 ± 1.8mW. Chronaxia was 13.9 ms. **C** However, a notable increase of the threshold energy was necessary at longer stimulus duration (slope 0.03 ± 0.0004 *p* < 0.01). This energy increase is explained by lateral heat diffusion and loss at longer laser stimuli

The 75 laser-sensitive neurons were investigated with laser stimuli of increasing energy by varying stimulus duration at three different powers (35, 60, 70mW; Fig. 5A). Threshold values concerning laser power and stimulus duration were fit by a hyperbola (Fig. 5B) and displayed a rheobase of 30.2 ± 1.8mW indicating the minimum laser power for the induction of a nociceptive laser-heat response, and a chronaxia of 13.9 ms. Threshold stimulus duration, i.e., duration to induce specific laser-heat responses in 50% of all neurons tested, for 35mW (78 ms) was nearly ten times higher than for 70mW (8 ms; Fig. 5B); if a constant amount of energy would have been required for cell activation, this difference should have been a linear factor of two, only. However, the energy necessary for specific heat responses markedly increased with increasing stimulus duration: The 50%-threshold energy at 78 ms (35mW) was four times higher (2.73 mJ) than that at 8 ms (70mW: 0.58 mJ; Fig. 5C). Thus, the stimulus energy increases with increasing duration, suggesting that relevant energy is lost by lateral heat diffusion the longer the stimuli are.

### Encoding of suprathreshold stimulus intensities

**Fig. 6:**
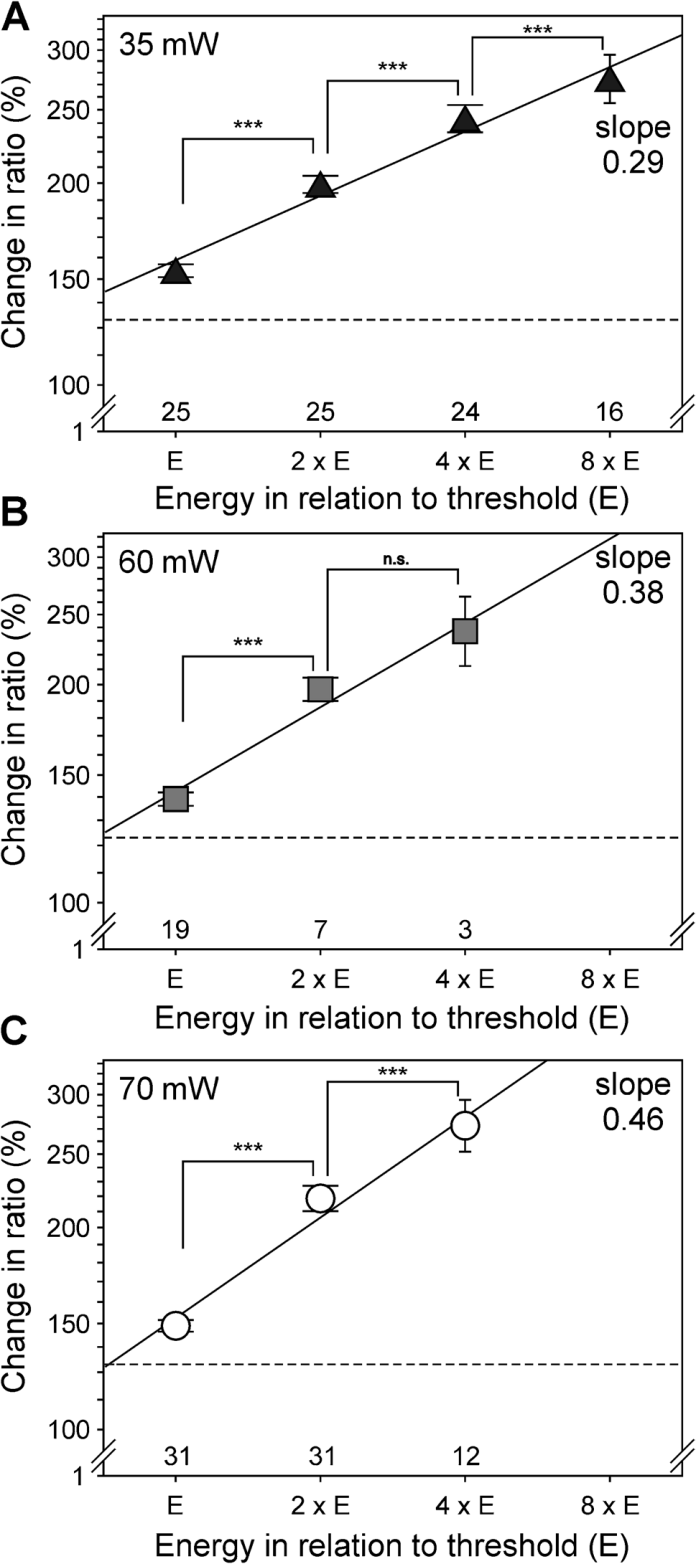
DRG neurons encode suprathreshold laser stimulus intensity. Mean peak amplitudes of calcium responses (‘change in ratio’ calculated as the relative increase of ratio signal above initial baseline) at the first suprathreshold energy (“E”) and multiples thereof at different laser power are shown: 35mW (**A**), 60mW (**B**), 70mW (**C**). Data are presented in a double logarithmic scale and the linear increase matches the exponents of a power function (Stevens exponents given as “slope”). Mean ± sem, minimum value for significant laser response is indicated as a dashed line; numbers of cells studied are given at the bottom of each graph; n.s. not significant, **p < 0.01, ***p < 0.001, Student’s paired t-tests

All 75 neurons sensitive to laser heat were stimulated with increasing energies after reaching individual threshold energy (“E”, Fig. 6); the duration of the laser pulse was doubled after each calcium transient (cf. Figure 3). For all three powers (35, 60, and 70mW), there was a significant encoding of stimulus intensity (see Fig. 6 for individual *p* values) but the increases in calcium transients became smaller with each successive doubling of stimulus duration (Fig. 6). The slopes in double logarithmic space were 0.29 at 35mW, 0.38 at 60mW, and 0.46 at 70mW, clearly demonstrating intensity coding but again energy loss by lateral heat diffusion at longer stimulus durations.

### Spatial mapping of heat sensitivity of the somata of nociceptive neurons

**Fig. 7:**
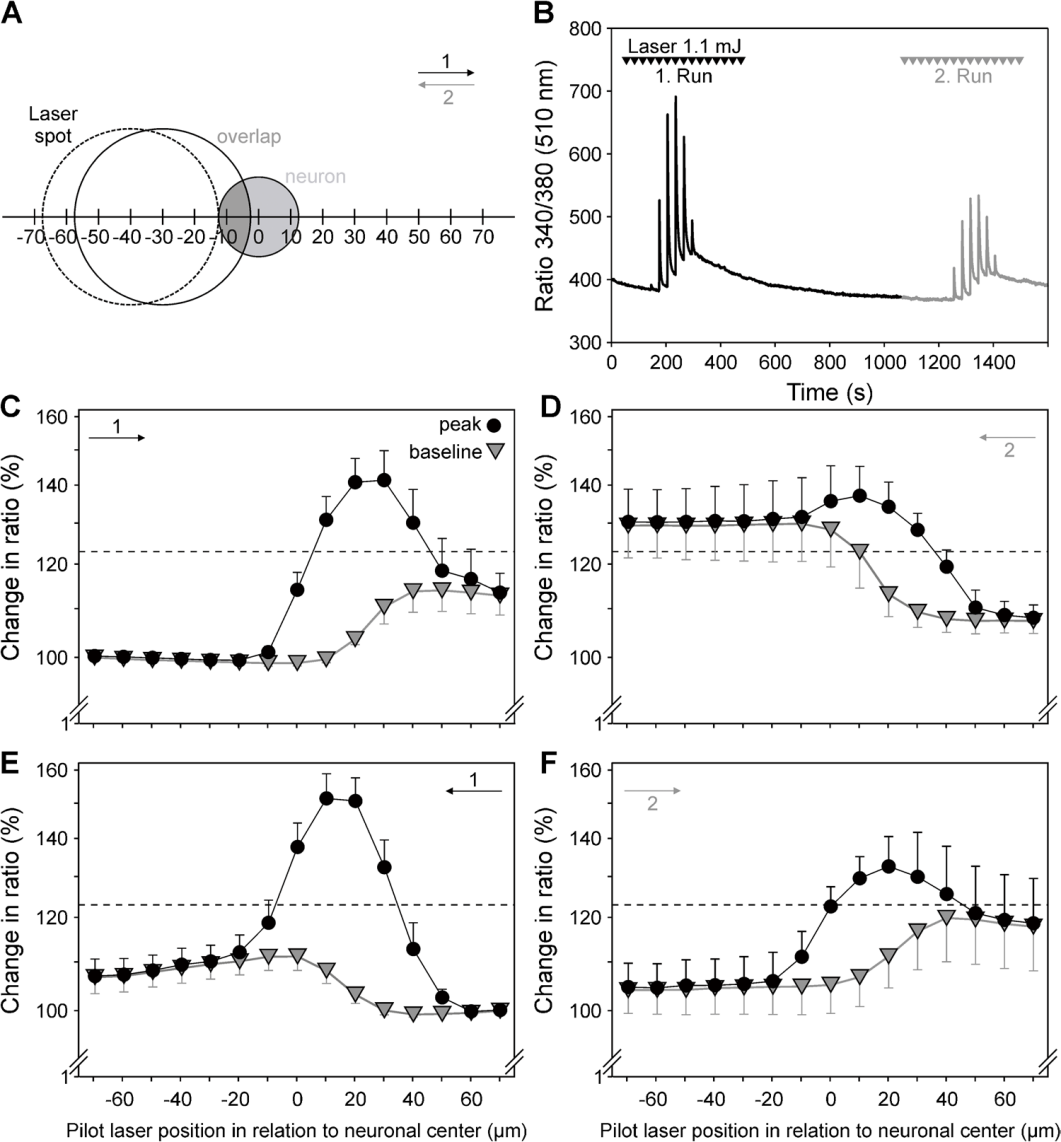
Spatial mapping of heat sensitivity of the somata of nociceptive neurons. **A** Schematic of the mapping procedure: the first laser pulse was applied at a distance of 70 µm away from the center of the neuron. At a position of − 30 µm an overlap between neurons (here *d* = 25 µm) and laser spot (*d* = 55 µm) was reached. This and the number of overlapping positions depended on the size of the neuron tested. The overlap (dark grey) between the two round areas (laser spot, large open circle, and neuron, light grey) was determined and the percentage of the cell area stimulated was calculated. **B** Representative single mapping experiment: When the laser spot was sequentially moved across a nociceptive neuron (*d* = 26.3 µm) from left to right, calcium transients in response to consecutive stimulation (arrowheads; ISI = 30 s) initially increased and then decreased as the laser moved beyond the neuron and the baseline also increased due to incomplete recovery. After a 10-min break, the laser was moved backward (right to left) along the same track. When the laser was first moved from left to right (**C**, “1,” *n* = 5) and then from right to left (**D**, “2”), all calcium transients in the second run were smaller and a tonic accumulation of calcium was seen. Maximum response occurred at x =  + 20 µm. When the laser was moved first from right to left (**E**, “1”) and then from left to right (**F**, “2,” *n* = 5), findings were similar and the maximum was also seen at x =  + 20 µm. The displaced maximum indicated a shift of the infrared laser spot in comparison to the pilot laser due to lower refraction at a longer wavelength (compare Fig. 1A). (C, D, E, and F: mean ± sem: filled circles: amplitude; grey triangles: baseline; relative “change in ratio” above initial baseline, as in Fig. 6)

**Fig. 8:**
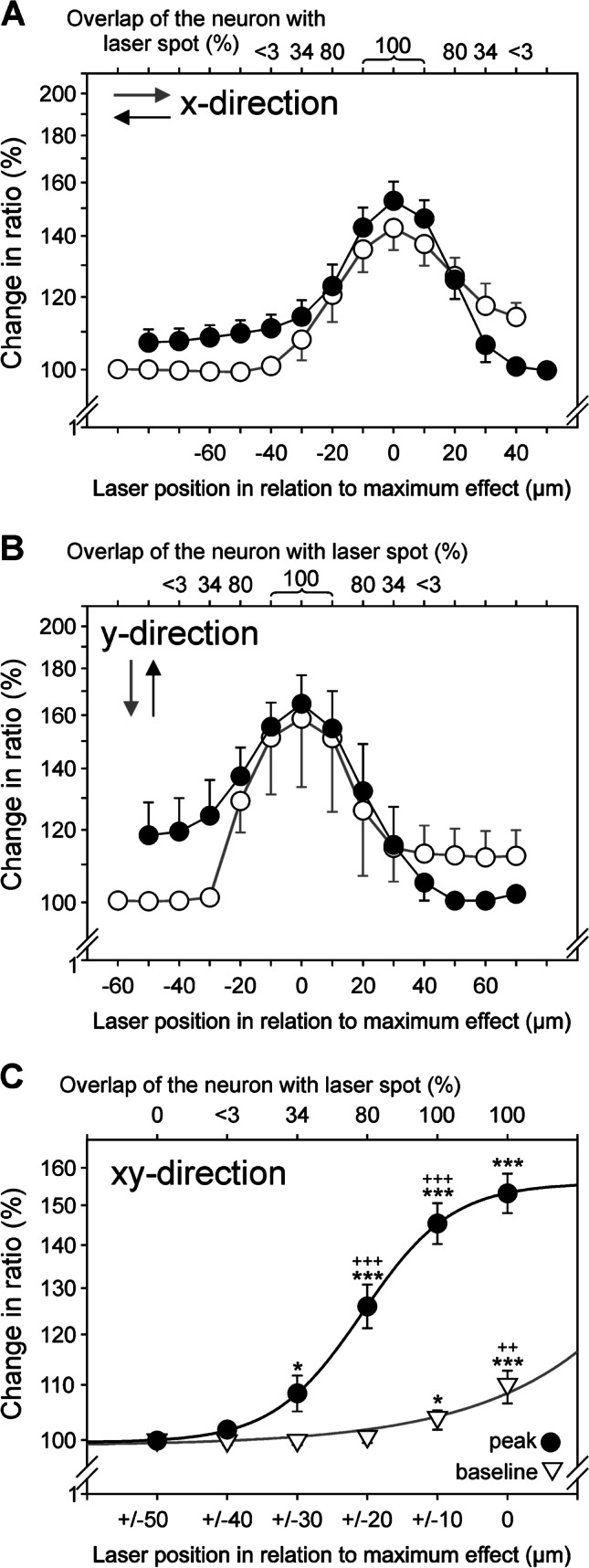
Heat responses of nociceptive neurons display spatial summation with increasing stimulated soma area. **A** Mean response sizes (peak amplitudes) to different overlaps of the laser with the cell soma in x-direction when the laser spot moved from left to right (open symbols) and right to left (filled symbols) (*n* = 5 for both directions). **B** Mean response sizes (peak amplitudes) in y-direction when the spot moved from above to below (open symbols; *n* = 4) and below to above (filled symbols; *n* = 2). Arrows mark the stimulus direction. Calcium transients were aligned to maximum effect, only calcium transients of the first run are shown. Note that there are three positions with 100% cell heating (marked as “100”), since the laser spot (55 µm) was larger than the neurons (< 32.5 µm). **C** Mean increase of fluorescence ratio 340/380 nm as a function of the percentage cell area heated (filled circles: amplitude; open triangles: baseline). Heating of at least 34% of the neuron leads to a significant calcium increase. A larger overlap between laser spot and neuron also leads to larger heat responses suggesting spatial summation within the cell soma. The maximum response occurred with the laser centered on the cell, this is explainable by the Gaussian beam profile of our laser (* *p* < 0.05, *** *p* < 0.001, ANOVA with LSD post hoc test versus first (0% overlap), ^+^ *p* < 0.05, ^++^ *p* < 0.01, ^+++^ *p* < 0.001 LSD post hoc test vs. preceding overlap, relative “change in ratio” above initial baseline, as in Fig. 6)

We scanned 16 laser heat-sensitive neurons with suprathreshold laser pulses (1.1 mJ, spot diameter 55 µm, see Fig. 7A) along two axes (X and Y) and in both directions along these axes. We intended to (1) identify which fraction of the neuron had to be heated to elicit laser-induced calcium transients, (2) look for evidence for spatial summation within the soma, and (3) checked how rapidly the calcium signals spread to unstimulated parts of the somata. Neurons displayed specific cellular calcium transients on four to six positions of the laser spot (Figs. 7B–F and 8). When the overlap between laser spot and neuron increased, amplitudes of calcium transients rose, and vice versa, baseline was, however, often not reached again after a pulse at an ISI of 30 s. Spatial mapping was repeated in the opposite direction after a few minutes’ break. On this second run, calcium transients were generally lower, independent of the starting direction (compare “1” and “2” in Fig. 7C, D as well as in E, F). Thus, only calcium transients of the first run were evaluated statistically.

In X-direction, the mean maximum responses were detected at a relative position + 20 µm lateral to the right side of the centered pilot laser spot, irrespective of whether the first run was from left to right (*n* = 5; Fig. 7C) or from right to left (*n* = 5; Fig. 7E). Thus, the infrared laser spot was slightly offset vs. the pilot laser spot, probably due to refraction in the cover slip that was hit at an angle of 76° (compare Fig. 1). As a matter of fact, no such offset was detected for mapping in Y-direction since there was no refraction in this direction (*n* = 2 starting from below, *n* = 4 starting from above).

In Fig. 8, all heat stimulus mapping data were adjusted to the maximum response amplitude indicating the alignment of the laser with the center of the neuron. Calcium transients were observed over a distance of 40–60 µm in both X- and Y-direction. These data indicate that a 100% overlap of the laser spot and cell surface was not necessary to elicit a specific calcium transient. One-way ANOVA revealed significant effects of overlap on both, peak amplitude (F_(5,90)_ = 41.25, *p* < 0.001) as well as prestimulus baseline (F_(5,90)_ = 7.96, *p* < 0.001). At an overlap of 3%, amplitudes did not differ as compared to the initial baseline, i.e., the mean calcium load before any stimulation (*p* = 0.64, LSD post hoc test), whereas 1/3 overlap (*p* < 0.05) or more (all *p* < 0.001) did induce significant calcium responses above baseline. Moreover, calcium transients significantly rose as a function of the heated area (Fig. 8C) indicating significant spatial summation.

### Calcium changes in unstimulated cell parts

**Fig. 9:**
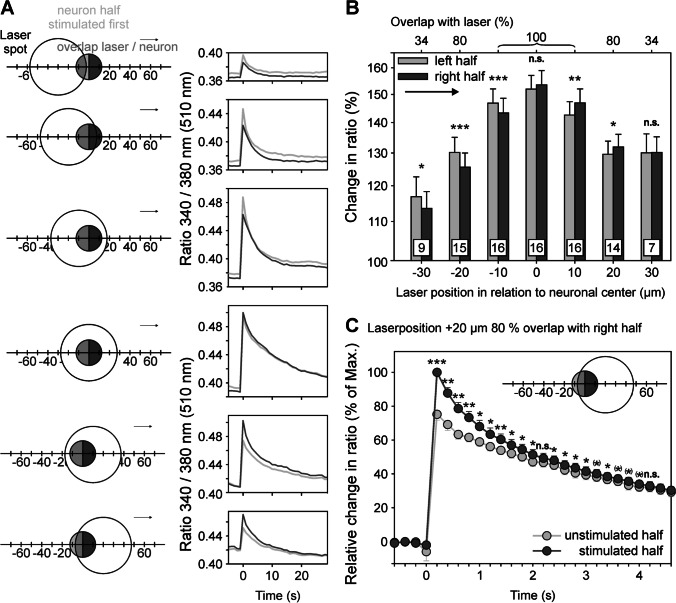
Local heating of subcellular parts leads to rapid intracellular calcium spreading beyond the stimulated area. **A** Representative mapping experiment in X-axis with separate analysis of the two neuronal halves (left half of neuron: light grey areas in the left column and light grey lines in the right column: right half; dark grey) at different overlaps of the laser spot (large open circle, diameter 55 µm) with the neuron (small circle, diameter 25 µm). Left column: spatial arrangement of laser relative to neuron, right column: calcium transients in the two halves of the neuron. When the laser is moved from left to right, only the left half (light grey) is stimulated but the unstimulated right half also responded with an albeit smaller calcium increase (dark grey); the area of the laser overlapping the neuron is given in medium grey. Only, when the laser is exactly centered (fourth trace from above) transients in both halves are exactly of the same size. When the laser was further moved rightwards, only the right half is stimulated (dark grey) but the unstimulated half also displayed a reduced transient (light grey). **B** The calcium response amplitudes at different overlaps of experiments confirmed that the stimulated half displayed significantly larger transients than the unstimulated half; number of laser-induced calcium transients investigated are given in squares, n.s. not significant, *p < 0.05, **p < 0.01, ***p < 0.001 Student’s paired t-test left versus right half, relative “change in ratio” above initial baseline, as in Fig. 6. **C **Statistical analysis of the time course of the transients from the stimulated cell half (dark grey) and unstimulated half (light grey; cf. inset) at a higher sampling rate (5 Hz; *n* = 6) revealed no difference in latency, but a larger response amplitude at the stimulated side; after 3 s the calcium signals of both cell halves converged suggesting that intracellular calcium gradient dissipated by diffusion (*n* = 6 cells). n.s. not significant (*) *p* < 0.1, *p < 0.05, ** *p* < 0.01, ***p < 0.001 Student’s paired t-test stimulated versus unstimulated

We then asked whether the laser heat-induced calcium transients are limited to the stimulated cell part or whether they spread to unstimulated sites. Therefore, analysis was separately performed for the stimulated and the unstimulated part of the neurons (Fig. 9). When coming from one side (i.e., from left in Fig. 9A), the stimulated half (light grey) displayed significantly larger responses than the unstimulated one that, however, also displayed a response (dark grey). When the laser is exactly centered (fourth trace in A from above), transients in both halves are exactly of the same size; please note that a 100% overlap of the laser with the neuron (indicated in middle grey) was reached at three successive positions (traces 3, 4, 5) but only at the second of those the laser was exactly centered above both halves. When the laser was further moved to the right, only the right half was stimulated (dark grey) but the unstimulated half displayed a reduced transient, again (light grey). Statistical analysis of the maximum calcium responses at different overlaps confirmed the observation that the stimulated half displayed significantly larger transients than the unstimulated (Fig. 9B).

In addition to 15 neurons that were investigated with a higher temporal resolution (5–25 Hz) of the recorded fluorescence signal, we attempted to determine the speed of the calcium spread from stimulated to unstimulated cell parts (cf. sketch in Fig. 9C). We found no difference of peak time of the unstimulated vs. the stimulated half of the neuron and there was also no difference in the beginning of the intracellular calcium rise detectable at the maximum acquisition rate available. Thus, calcium signals spread throughout the soma, but at a speed beyond our temporal resolution (up to 25 Hz sampling rate, i.e., faster than 330 µm s^−1^ for the smallest cell investigated [diameter 26.5 µm]). These experiments revealed that calcium increased nearly simultaneously in both cell halves, but there was a significant difference in the amplitude between the stimulated and the partially stimulated cell half for up to 3 s after stimulation (Fig. 9C). After 3 s, the calcium signals of both cell halves converged suggesting that the intracellular calcium gradient dissipated by diffusion [2].

## Discussion

This study shows that infrared lasers can be used to investigate heat transduction mechanisms in native sensory neurons at high temporal and spatial resolution. We now used calcium imaging instead of electrophysiology—a method providing spatial resolution of neuronal excitation [28]. Short intense stimuli (8 ms, 70mW) were sufficient to activate heat transduction, longer pulses required lower threshold power following a hyperbolic function with a rheobase of 30mW. Laser-heat responses in DRG neurons depended upon activation of TRPV1 [4] as they were reduced (long) or fully blocked (short pulses) by the competitive antagonist CPZ. Regarding temporal summation, DRGs were capable of linearly encoding laser intensity in double logarithmic scaling with exponents of about ≤ 0.5 at a given laser power. We have previously shown that the short laser pulses are around the heat pain threshold in humans whereas the long-lasting are clearly painful [28]. Spatial summation characteristics were demonstrated by stimulating restricted cell areas; heat responses were induced when at least one-third of the neuron was heated and further increased with the stimulated cell area. Calcium transients seen in unstimulated parts of a neuron were due to depolarization by heat transduction and, presumably, heat-activated action potential discharges and concomitant opening of voltage-gated calcium channels, and later equilibrium by passive diffusion of calcium.

### Rapid activation of native sensory neurons in live-cell imaging by direct laser heating

Heat stimulation of native cells using laser pulses is hindered by the fact that the radiation has to pass microscopes optics and water with ideally minimal absorption and/or deflection at relevant boundaries, but should be absorbed maximally by cells themselves. Therefore, we used a diode laser stimulator that focused 1475 nm radiation—that displays a relative absorption peak in water (dermal penetration depth 435 µm; [24, 25, 28, 43–45])—directly to the nociceptive neurons adherent upside down to a cover slip for precisely localized heat stimulation without having to pass through microscopes’ optics and/or extracellular solution. We achieved rapidly increasing heat pulses directly at a neuron, i.e., heat stimulation with high temporal control. The shortest stimulus duration that elicited specific responses in our experiments was 4 ms (70mW) which is compatible with the occurrence of laser-induced *I*_heat_ in TRPV1-expressing cells “within a few milliseconds” [44] and significantly faster than any chemical activation [45]. Upon termination of the laser pulse, the temperature returned to baseline even more rapidly by passive cooling than it increased upon active heating. These observations are in agreement with previous experiments [11, 44, 45]) and theoretical simulations [44]. The small heated volumes are tremendously cooled by the surrounding fluid upon the termination of laser radiation clearly indicating that all subsequent observations do not reflect any non-specific temperature dependence.

Defining specific thresholds for thermal sensitivity is hampered by the fact that all processes display affection by changing temperature—may they result from specific heat activation or represent non-specific artefacts of heating. We defined a specific neuronal response as a signal beyond the size and duration of the heat sensitivity of the dye. Overall, 89% of the small neurons were laser-sensitive, a percentage within the range reported previously (56–93%; [5, 7, 9, 17, 18, 23, 41]). A relatively high proportion of laser-sensitive neurons is further compatible with findings that the threshold of *I*_heat_ decreases with increasing heat slopes [11], a phenomenon also known in vivo [35].

Regarding the peak amplitude, laser responses were reduced or fully blocked by CPZ, and several of the neurons under investigation showed a remarkably high pharmacological reduction of laser responses (≤ 90%), a value similar to that described for those using a 980 nm laser with HEK cells expressing TRPV1 [16]. An incomplete pharmacological reduction of heat responses in native DRGs has already been noted before [9, 10, 18, 20, 23]. These results are in accordance with our recent findings that TRPV1 is mainly activated by non-damaging laser heat [28] whereas additional mechanisms (cf. [40]) are co-expressed with TRPV1 in nociceptive neurons [14, 21]. These may act in concert with TRPV1 to account for the transduction of more intense stimuli of longer duration [28]—as suggested by the two different heat transduction mechanisms found in nociceptive afferents [38, 39].

### Strength duration properties and laser heat intensity coding in native DRG neurons

Mean utilization time was 8 ms at high intense pulses that non-linearly increased by a factor of 8 when laser power was reduced by a factor of two, only, following a hyperbolic function—similar to TRPV1-expressing HEK293 cells [28]. This finding further indicates that the longer a radiant stimulus is applied the more thermal energy is lost via lateral temperature diffusion (cf. [28, 44];).

Suprathreshold stimuli induced significantly stronger calcium transients. The maximum slopes of our stimulus–response functions were around 0.5 in double log scales (i.e., Stevens exponents, [1]). Accordingly, brief cutaneous CO_2_-laser stimuli applied to human skin in vivo also displayed Stevens’ exponents below 1 (0.59–0.64; [33]) as did calcium responses in TRPV1 expressing HEK293 cells [28]. When our laser was used to elicit human heat pain, Stevens’ exponents were somewhat steeper (exponents of 1.1 to 1.22, [28]). Responses in our cell experiments were obtained at relatively short ISIs known to induce marked fatigue of the nociceptive responses upon repeated stimulation of the same cell [31] whereas this effect is usually avoided in vivo by shifting the heat stimulator after each stimulus [8]. Thus, those differences may at least in part be explained by the peripheral nociceptor fatigue induced in vitro. Differences between neuronal monolayers in a dish here with three-dimensional structures in vivo as well as between lasers of different wavelengths may also be explained, however, by differing penetration depths and spatial summation: whereas CO_2_-laser stimuli are absorbed in uppermost layers and reach deeper layers via passive heat conduction, near-infrared lasers are capable of exciting more than one neuron at a time since they actively heat the entire volume under irradiation due to its penetration peaking at around 280 μm tissue depth in human skin [28]. This observation may prefer near-infrared laser radiation for heat pain induction.

### Spatial summation and spatial coding of laser responses

The response magnitude at suprathreshold laser intensities of cutaneous C-fiber nociceptive afferents depended on the percentage of the receptive-field heated [37]. Up to now, the effects of partial laser-heating of single neurons could not be investigated; our setup now allowed for the first time precise spatial control of isolated microscopic neuronal laser stimulation. Heating ^1^/_3_ of the area of a neuron was sufficient to induce calcium transients in nociceptive neurons, transients significantly increased with increasing the stimulated area giving a peak response when the laser was centered; thus, we now for the first time demonstrate the phenomenon of spatial summation within a single nociceptive neuron. This observation resembles spatial summation known from sensory physiology, e.g., in response to increasing skin areas stimulated with a laser by increasing the number of stimulated free nerve endings within one or more receptive fields [32, 37].

The laser-induced calcium responses were, however, not locally restricted to the heated area but rapidly spread to unheated parts of the cell. The speed of intracellular spreading of the calcium signal was always above the temporal resolution of our system (max. 25 Hz, corresponding to 40 ms between successive images, which translates into a speed above 330 µm s^−1^). This is a known problem when investigating calcium transients with fluorescence [3]. The speed of diffusion of free calcium in cytoplasma is relatively slow: Intracellular second messenger waves propagate by diffusion at 5–100 µm s^−1^ [6, 22]; using FURA-2 the velocity of calcium waves in Purkinje neurons was estimated to be 30 µm s^−1^ [15, 34]—while action potentials propagate at velocities above 1,000,000 µm s^−1^. Therefore, the predominant mechanism of heat-induced calcium signals in unstimulated parts of the neurons depended on depolarisation and action potential discharges activating voltage-gated calcium channels [9, 12]. Nonetheless, a small portion of the heat-induced neuronal calcium transients was mediated by direct activation of heat-transducer molecules [40], because calcium signals were stronger at the irradiated halves than in the non-irradiated parts of the cell. The difference between the heat-stimulated and the unstimulated neuronal halves disappeared within 3 s; if the cell is assumed as a circle (diameter 25 µm, area ~ 500µm^2^), the duration of 2–3 s is compatible with calcium spread by diffusion to level intracellular calcium differences thereafter.

### Limitations

Due to its outstanding temporal resolution and the possibility to clamp membrane potentials for the study of voltage gating, electrophysiology is the ideal technique to study neuronal excitability including rapid temporal aspects of heat transduction (please refer to [11] and [45]). However, this technique does not allow to investigate spatial phenomena such as differences between stimulated and unstimulated parts of the soma. We thus used calcium imaging as an indirect measure of neuronal excitability, because it provides outstanding spatial resolution [28]. As a trade-off, we had to accept the limited temporal resolution, hampering the analysis of the speed of spread and the contribution of voltage-gated calcium channels as discussed above.

Furthermore, we did not calibrate the observed calcium fluorescence responses induced by laser-heat due to the marked thermosensitivity of this calibration procedure itself and did not normalize response patterns between cells because we were mainly interested in relative and not absolute changes in intracellular calcium induced by heat. We could not systematically determine calcium response sizes and their changes with time, i.e., by calculating the “area under the curves” defined by our ratio traces after short-lasting laser stimulation, due to incomplete recovery, repeated stimulation, accumulation, and release of intracellular calcium from internal stores such as mitochondria [42], etc. and therefore cannot exclude that additional slow- and long-lasting processes independent of TRPV1 may slightly contribute to laser-heat responses, as well.

Spatial integration of heat-induced signals would be most interesting to study within the epidermis or under culture conditions that replicate the intraepidermal nerve branches and terminals. However, cell culture usually gives rise to random overlapping networks of neurites and overgrowth by fibroblasts when using native long-term cultures. Therefore, we used the neuronal somata as models for their own axons, axon branches, and terminal. While the soma has a similar expression of membrane proteins, the surface-to-volume ratio and hence electrotonic and diffusion conditions are different. However, we were able to identify a contribution of diffusion to the modulation of calcium homeostasis in spite of these limitations.

Last but not least, we did not address potential sex-related differences in spatial integration of heat-induced calcium signaling. While females are known to be more heat-sensitive on a behavioral level than males [27], CNS signal processing of emotional and cognitive aspects of pain appears to play the dominant role in these differences [29] and there is so far no evidence that spatial signaling may be different. Since we included tissue from both female and male rats, one may regard our results as being gender-neutral.

### Conclusions

In conclusion, NIR laser pulses induced laser-heat responses in native rat nociceptors with complex dose–response properties that linearly encode the applied suprathreshold stimulus energy in double logarithmic space. These calcium transients were elicited by activation of heat transduction channels—at least in part TRPV1—activating in turn voltage-gated calcium channels as concluded from the observed properties of heat-induced intracellular calcium spread after laser stimulation. For the first time, spatial summation could be demonstrated at (sub)cellular level by heating varying proportions of a neuron, i.e., between 30 and 100% of the DRG profile, at unprecedentedly high spatial resolution.

## Authorship policy

Elisabeth Jubileum, phone: +49 (0)6131-37812321, email: e.jubileum@rfk.landeskrankenhaus.de

Conceived of or designed study, performed research, analyzed data, and wrote the paper.

Uta Binzen, PhD, phone: +49 (0)621 383 71860, email: ta.binzen@medma.uni-heidelberg.de

Conceived of or designed study, analyzed data, and revised the paper.

Rolf-Detlef Treede, MD, phone: +49 (0)621 383 71400, email: rolf-detlef.treede@medma.uni-heidelberg.de

Conceived of or designed study, analyzed data, and revised the paper.

Wolfgang Greffrath (MD, corresponding author), phone: +49 (0)621 383 71412, email: wolfgang.greffrath@medma.uni-heidelberg.de

Conceived of or designed study, performed research, analyzed data, and wrote the paper.
